# The Importance of Understanding Deep Learning

**DOI:** 10.1007/s10670-022-00605-y

**Published:** 2022-08-07

**Authors:** Tim Räz, Claus Beisbart

**Affiliations:** 1 University of Bern, Institute of Philosophy, Länggassstrasse 49a, 3012 Bern, Switzerland; 2 Center for Artificial Intelligence in Medicine, University of Bern, Bern, Switzerland

## Abstract

Some machine learning models, in particular deep neural networks (DNNs), are not very well understood; nevertheless, they are frequently used in science. Does this lack of understanding pose a problem for using DNNs to understand empirical phenomena? Emily Sullivan has recently argued that understanding with DNNs is not limited by our lack of understanding of DNNs themselves. In the present paper, we will argue, *contra* Sullivan, that our current lack of understanding of DNNs does limit our ability to understand with DNNs. Sullivan’s claim hinges on which notion of understanding is at play. If we employ a weak notion of understanding, then her claim is tenable, but rather weak. If, however, we employ a strong notion of understanding, particularly explanatory understanding, then her claim is not tenable.

## Introduction

The increasing use of machine learning, in particular of deep neural networks (DNNs), is often met with suspicion. One concern is that deep learning algorithms are not well understood. For instance, we do currently not have an explanation of the empirical fact that many DNNs are predictively successful in application.^1^ Our lack of understanding DNNs is particularly relevant when they are used in science. One of the main tasks of science is to help us understand the world. But how can science do so if its own methods are not well understood?

In a recent paper, Emily Sullivan (2022) has taken a novel and surprising perspective on this question. She argues that if we use DNNs to understand phenomena in the world, and if we fail to understand these phenomena, this is not due our deficient understanding of DNNs. Rather, what limits our understanding of the phenomena is the low degree to which we can link the DNNs to the phenomena that are investigated.^2^

In this paper, we will argue against Sullivan’s claim and for the common view that our current lack of understanding of DNNs does indeed constrain our understanding of phenomena investigated with DNNs. Our arguments are based on recent results from computer science. In particular, we argue that the predictive success of DNNs is a brute, contingent fact, and not something we understand theoretically. This, in turn, threatens our understanding of phenomena when we apply DNNs in science.

In Sect. 2, we briefly reconstruct Sullivan’s arguments. In Sect. 3, we argue that DNNs do not implement a simple, known function, as maintained by Sullivan. In Sect. 4, we cast doubt on the idea that so-called saliency maps contribute much to a general understanding of DNNs. In Sect. 5, we argue that Sullivan’s main claim critically hinges on which notion of understanding is at play. If we employ a weak notion of understanding, e.g. meaning some degree of objectual understanding, then her claim is tenable, but rather weak. If, however, we employ a stronger notion of understanding, in particular explanatory understanding, then her claim is not tenable. We conclude in Sect. 6.

## Sullivan’s Arguments Reconstructed

### Sullivan’s Theses

Sullivan distinguishes between two different kinds of understanding. First, there is the understanding *of* a machine learning (ML) algorithm or model.^3^ Second, there is the understanding of phenomena *with* an ML model, or *using* an ML model as a vehicle of understanding, as one may put it. Sullivan is interested in the relation between these two kinds of understanding: How does a lack of understanding *of* an ML model affect our ability to understand phenomena *with* that model?

When we try to understand phenomena using ML models, we may fail. Sullivan identifies two possible reasons for such a failure. The first possible reason is “link uncertainty”, i.e., we lack evidence (and thus knowledge) about how a model and its output are linked to the phenomena under consideration. The second possible reason is that we lack understanding of how the model itself works. Sullivan claims that while the first possible reason is in fact an issue, the second is not. Thus, Sullivan argues for the following two theses:

1. **Negative Thesis:** If we want to understand phenomena using ML models, it is not necessary to understand how ML models work better than we currently do.
2. **Positive Thesis:** If we want to understand phenomena using ML models, the models must be suitably linked to the phenomena in question.^4^

The meaning of both theses depends on the notions of understanding presupposed by Sullivan, although she does not endorse a particular notion of understanding: “My arguments do not so much trade on any positive notion of what understanding and explanation is” (p. 111). But she assumes a close connection between understanding and explanation: “[i]n a slogan: explaining why helps us to understand why” (p. 110). We thus take it that her main interest is in explanatory understanding, also called understanding-why (see e.g. Baumberger et al., 2017 for this notion). Below we will spell out in more detail what “understanding” means for Sullivan and how this affects the meaning and the plausibility of the Negative Thesis.

A challenge to any precise formulation of both theses is the fact that understanding comes in degrees. Criticism of the Negative Thesis easily becomes trivial if this thesis is understood as denying that understanding an ML model is necessary for *maximal* understanding with the model. In what follows, we do not assume that the Negative Thesis is about maximal understanding.^5^

In Sullivan’s argument for the Positive Thesis, she compares three examples of understanding with ML models, pointing out that the degree to which the models provide understanding differs in accordance with their differences in link uncertainty. We will not discuss this argument in detail, because the Positive Thesis is not the target of our criticism. Note, however, that even if the three case studies support the Positive Thesis, they do not thereby support the Negative Thesis, because even if link uncertainty is relevant for understanding phenomena using DNNs, this does not show that understanding DNNs is irrelevant for understanding phenomena using DNNs.

### DNNs are Implementation Black Boxes

If there are parts of a model that we do not know and therefore do not understand, then, according to Sullivan, this part of the model is *black-boxed*. *Prima facie,* models that are partially or entirely black-boxed seem problematic when we want to use them as vehicles of understanding. But Sullivan argues that so-called *implementation black boxes* need not stand in the way of understanding phenomena using a model. An implementation black box occurs when we do not know how a part of a model is implemented.

In one hypothetical example, Sullivan assumes that a climate model (or the related simulation algorithm) needs to compute factorials, i.e., the function $f:{\mathbb {N}} \rightarrow {\mathbb {N}}$, $f(n) = n!$. Factorials can be implemented in several, computationally different ways. However, she argues, we do not need to understand the implementation of the factorial function used in the climate model in order to understand the target system using the model; it is sufficient to know that the factorial was implemented correctly. In this case, the implementation black box is harmless.

What about higher-level black boxes? Sullivan argues that, under certain conditions, higher-level aspects of models are harmless implementation black boxes as well. As long as we know which abstract function is computed by a model, this does not impede our understanding of the target system, even if the entire model is black-boxed. Sullivan proposes that this also applies to other cases, arguing that a simulation of Schelling’s famous model of segregation is a harmless kind of implementation black box, and that the same argument may also apply to DNNs.

At this point, the key issue is the strength of the analogy between DNNs and factorials. We will argue in Sect. 3 below that this analogy is weak.

### Understanding DNNs Beyond Implementation

Sullivan argues that implementation black boxes may be harmless; at the same time, she seems to grant that there are aspects of DNNs that we do not understand, and that this lack of understanding may be different in kind from implementation black boxes. For example, we may not know the parameter values of a trained DNN before we actually train it, and we may not know how to interpret the resulting parameter values.

Why does our lack of knowledge not cause problems when we use DNNs as vehicles? Sullivan argues that the aspects of DNNs we do not understand do not matter for our use of DNNs as vehicles, because our knowledge is nevertheless sufficient. She writes:

[...] the modeller relies on a wealth of knowledge and research about what methods to follow to build a generalizable model for the task at hand. [... the modelling process] is not back-boxed [sic!] at the highest level, such that it would prevent understanding of the phenomenon the resulting model aims to capture. (p. 122)

Sullivan’s justification of this claim is that we have two kinds of knowledge that provide sufficient understanding of high-level properties of DNNs. First, we have “a general idea of how the finalized model works in virtue of having knowledge about how the model was trained and validated” (p. 122). Presumably, this knowledge is related to statistical results about the generalization properties of ML models; see Sullivan’s footnotes 9 and 10. We will discuss what is currently known about generalization properties in Sect. 5.2.

Second, we have what Sullivan calls “indirect means”, of which saliency maps, used in image classification, are one example. Given a trained classifier and an input image to be classified, a saliency map highlights which pixels of the input image are relevant for its classification by the model. Saliency maps can be used as a diagnostic tool, for example to detect whether a model places undue weight on a part of the image that should be irrelevant. How much understanding do saliency maps provide? Sullivan writes that while saliency maps are approximative and do not reveal every kind of dependency, “different types of saliency testing are enough to satisfy our need to know the high level details of how the model works to open the door to understanding the phenomenon the model bears on.” (p. 122) We will critically assess the ability of saliency maps to provide understanding in Sect. 4.

### Understanding with DNNs

In order to make the point that we can indeed understand with DNNs as vehicles, Sullivan discusses several cases where we are able to gain scientific insights despite the fact that we do not understand certain aspects of DNNs. But such insights are only relevant to the Negative Thesis if they constitute understanding phenomena using DNNs. We thus need to discuss whether this condition is met. Here we identify three epistemic achievements that are stressed by Sullivan and might constitute understanding phenomena using DNNs.

First, Sullivan emphasizes the *predictive accuracy* of DNNs. One of her case studies is the so-called deep patient model, which can be used for disease prediction and other purposes. Sullivan notes that the classification by the DNNs agrees with the available evidence to a high degree, and writes: “Simply having a highly predictive model, and knowing the high-level emerging properties of the model, uncovers that it is possible to use a machine learning representation for disease prediction” (p. 123). Thus, predictive accuracy might be one of the epistemic achievements leading to understanding.^6^

Second, DNNs may provide *how-possibly explanations*, that is, they may specify possible causes of diseases. Sullivan writes that a how-possibly explanation “simply highlights a possibility concerning the causes or dependencies of some phenomenon; it falls short of explaining how the target phenomena actually is caused or the actual dependences concerning the phenomenon” (p. 123).^7^

Third, DNNs may point researchers in the right direction for further investigations – they may play a *heuristic role*. Even if DNNs do not provide how-actually explanations themselves, they may contribute to finding how-actually explanations.^8^ This role of DNNs is emphasized in the following quote, in which Sullivan discusses a model for melanoma prediction:

The model can help physicians gain understanding about why certain medical interventions are relevant, and using the model can help explain medical interventions to patients. Moreover, the model can discover new visual patterns that are highly correlated with health or disease. This can further understanding, especially once these newly discovered patterns undergo further investigation. (p. 126)

We will discuss to what extent these three features do in fact contribute to understanding in Sect. 5.1 below.

## Objection 1: DNNs are not Implementation Black Boxes

We now turn to our criticism of Sullivan’s argument. We start with her point that DNNs are harmless implementation black boxes, in analogy to different implementations of the factorial function, which do not appear to stand in the way of understanding a target system. Here we argue that DNNs are not analogous to factorials.

To see why, let us consider how DNNs work. One of the benchmark problems of supervised learning is the classification of handwritten digits based on the MNIST dataset.^9^ The task is as follows: Given a 28$\times$28 greyscale image of a handwritten digit, find the correct classification of the image. A classifier is a function $f:X \rightarrow Y$, $f(x) = \hat{y}$, where *X* encodes the data to be classified, *Y* encodes the set of classes, and $\hat{y}$ is *f*’s prediction. Here, we simplify MNIST and take *X* to be the set of all 28$\times$28 black-and-white pictures, i.e., each pixel can only be either black or white. *Y* is the set $Y = \{0,1, ..., 9\}$. A classifier is correct if the classifier’s output $\hat{y}$ agrees with our intuitive judgment (or expert judgment) *y* about which digit is depicted in *x*, i.e., whether $y = \hat{y}$.^10^ A good classifier is a classifier that is correct in a large proportion of instances.

DNNs are very good at solving this type of classification problem. We initialize a DNN, with prediction function $\hat{f}$, with random weights; at this point it would not be a good classifier. Now we train $\hat{f}$. The first part of the dataset consists of 60,000 images of handwritten digits, $x_i$, together with labels $y_i$, indicating which digit is depicted. Abstractly, the training set is $X = \{ (x_i, y_i), i = 0... 60'000\}$. Training $\hat{f}$ on *X* means that $\hat{f}$ adjusts weights according to stochastic gradient descent. After training, we can test how well $\hat{f}$ classifies on a test set $X' = \{ (x'_i, y'_i), i = 0... 10'000\}$, which is also part of the MNIST dataset. Importantly, the examples in the test set are not in the training set. When we say that a DNN is good at solving the MNIST problem, we mean that $\hat{f}$ is good at classifying the pictures in the test set.^11^ This is what computer scientists mean when they say that DNNs generalize well on MNIST. Of course, DNNs also generalize well on problems that are much bigger and complex than MNIST.

Let us now return to Sullivan’s point that if we know the function computed by a model, for example the factorial, then we do not need to know how the model implements the computation, as long as the implementation is correct. The preceding account of how DNNs solve MNIST indicates that DNNs do not implement a known function in the same way in which a certain algorithm or a subroutine in a computer program implements the factorial. In more detail, there are two candidate functions that could be said to be implemented by the DNN, viz. *f* and $\hat{f}$. Consider first *f*, i.e., the “true” classifier, the function that classifies all images correctly. However, it is just not the case that we know *f* in the same way we know the factorial function. In the case of the factorial, we have a simple, short description of how to obtain the output for every possible input.^12^ In the case of the “true” classifier *f*, we have an explicit representation of only a very small portion of *f*, viz., for the training and test sets. Of course, we could expand the training and test sets, but doing this until we know *f* essentially amounts to going through all possible 28$\times$28 black-and-white images and determining which digit, if any, is depicted, which is practically impossible. There are $2^{28\times 28}$ possible black-and-white images of this size; in decimals, this is about $10^{236}$.^13^

Consider now $\hat{f}$. First of all, it is not clear whether, strictly speaking, the DNN can be considered to be an implementation of $\hat{f}$ because $\hat{f}$ is the function that maps inputs to outputs in the same way as the DNN. It is the input-output profile of the DNN, i.e., a property of it, and not implemented by it. Second, even if we granted that $\hat{f}$ were implemented by a DNN, $\hat{f}$ is not known in the way in which the factorial is known because $\hat{f}$ is extremely complex. Researchers sometimes cannot anticipate how $\hat{f}$ changes if the input is slightly modified. All typical users know is that $\hat{f}$ approximates the true classifier *f*, but since *f* is not known, this does not help either. Using the terminology recently proposed in Creel (2020), DNNs lack “functional transparency”, i.e., we do not know which algorithm is instantiated by a particular DNN.

As indicated above, Sullivan uses a second example, Schelling’s checkerboard model of segregation, as an analogy to DNNs. Her main points about Schelling’s models are as follows: First, for some time, it was unclear whether the model provides a how-actually explanation of racial segregation, because there was a lot of link uncertainty. Second, the implementation of the model was irrelevant for the question as to what extent the model can help us to understand racial segregation. *Per analogiam*, these two points are supposed to carry over to DNNs.

We agree with the first point about link uncertainty regarding both Schelling’s model and DNNs, but this point is only relevant to the Positive Thesis, which is not our focus. However, we think that the Schelling model and DNNs are relevantly disanalogous when it comes to understanding the model itself, which is relevant for the Negative Thesis. It is true that the implementation of the Schelling model does not matter much. However, the Schelling model is different from DNNs in having fixed dynamics, i.e., fixed, predetermined rules of transition, which are chosen by the modelers. The situation is very different with DNNs, where the dynamics are learned during the training process and not known by the modelers. Even worse, the modeling assumptions implicit in a DNN are not easily stated or summarized because they are too complicated and involve too many parameters. The assumptions underlying the Schelling model, by contrast, are much simpler. They are in fact a straightforward operationalization of a simple and intuitive story: People tend to move elsewhere when their ethnic group is clearly in the minority (see Hartmann, 1999 for models and stories). As a consequence, scientists can to some extent reason about the behavior of the model. This point does not hold true with regard to DNNs, which largely remain black boxes.

This does not mean that researchers do not have any idea how DNNs function. For instance, Buckner (2018, 2019) has suggested that we can explain the classifications of some DNNs with so-called “transformational abstraction”. While this may provide a kind of story about how DNNs function, the details of the model are too complicated and do not allow humans to reason with it. And the details indeed matter. If the functions used in a DNN’s computation are not implemented in an efficient manner, then computations will take longer, which limits our ability to use DNNs in application. The same goes for which exact activation functions we use, which exact optimization procedure, and so on. These are important issues to consider, but they are problems that we have on top of the fundamental difficulty that we know little about what function is computed by an DNN.

## Objection 2: No (Global) Understanding of DNNs from Saliency Maps

After the argument about implementation black boxes, Sullivan seems to grant that we may not fully understand DNNs. However, she argues, we have some insight into their working, and this is sufficient for understanding with DNNs. In particular, she singles out saliency maps as a method which helps us by “determining the suitability of the model” (p. 122), that is, by establishing that the model itself works properly. We will now argue that saliency maps do not establish this, for two reasons.

The first reason is that methods such as saliency maps provide very limited insight into the general working of a model. They are geared toward understanding the classification of particular instances by a model, not toward understanding more global properties of the model, which would require that these methods tell us about how a model behaves for many inputs. In computer science parlance, they provide local explanations.^14^ Recall that a saliency map can be interpreted as a heat map, which highlights regions of an image that are relevant to the classification of the image. Mathematically, a saliency map is the gradient of the model’s output (prediction) with respect to one input, that is, a map that measures how much the output would change if the components of an input image changed. This means that saliency maps only give us local and linear information. They tell us how a linear approximation of $\hat{f}$ behaves in a small neighborhood of the instance, and may differ significantly even for inputs that appear to be very similar.

It might be thought that researchers can use a sample of local explanations obtained using saliency maps to inductively infer how DNNs work more generally. However, to reliably infer explanations for a larger part of the input space, a huge number of saliency maps may be needed, depending on the nature and non-linearity of the predictor, which makes this approach unfeasible. Furthermore, this inductive approach would only work under the assumption that saliency maps for particular instances are reliable. This brings us to our second point.

The second reason for being cautious about the use of saliency maps is that it has been contested whether they can provide much insight into the classification even of particular instances. For example, Adebayo et al. (2018) critically examine saliency methods (which encompass saliency maps). They find that there is a lack of standards for assessing saliency methods, i.e., it is not clear under what criteria we should consider such methods to be reliable. Then the authors propose two criteria, randomization tests, one for the model and one for the data. The idea is that a saliency method should depend both on the model’s learned parameter values and on the training data; if the result of a method is independent of model and data, e.g., if the saliency method works similarly on a scrambled and an unscrambled version of a model, then the method should be discarded. Finally, the authors apply these tests to a number of saliency methods and find that some widely used saliency methods fail the two tests. In the conclusion, the authors write: “Our results show that visual inspection of explanations alone can favor methods that may provide compelling pictures, but lack sensitivity to the model and the data generating process” (p. 9).

To be fair, Sullivan grants that saliency maps do not provide a full understanding of a model. However, it is important to realize just how little saliency maps tell us about $\hat{f}$. This is compatible with Sullivan’s point that, in some cases, saliency maps and similar methods can pick up unintended features of a model, e.g., that the model relies on proxies for classification instead of the intended features (Sullivan 2022, p. 122). Saliency maps can indeed be useful. However, there is no guarantee that if a saliency maps looks fine, the model is fine. Saliency maps are heuristic tools; they do not provide general understanding of a model.^15^

## Objection 3: Understanding DNNs Matters

### Criteria for Understanding with DNNs

Our main argument against the Negative Thesis is that, on plausible readings of the notion of understanding with DNNs, the Negative Thesis comes out wrong.

Our argument is premised on the assumption, defended above, that Sullivan is interested in explanatory understanding using ML models. Several views of explanatory understanding are available. According to de Regt (2017, p. 92) we have explanatory understanding of a system if we can explain it using a theory that is intelligible to us and if the explanation is consistent and empirically adequate. Some authors do not require full knowledge of an explanation for some degree of understanding-why. Khalifa (2017, p. 14) proposes that the degree to which a person understands why p depends on how close her grasp of the explanatory nexus comes to knowledge of an explanation. Still, to understand why p is the case, people have to get close to a (correct) explanation.

In what follows, we will not work with any specific view of explanatory understanding though. Rather, we will go through the three epistemic achievements we have extracted from Sullivan’s paper in Sect. 2.4. For each achievement, we will discuss how close it comes to explanatory understanding with DNNs, to what extent DNNs realize this achievement, and what this means for the Negative Thesis. We will obtain three readings of the Negative Thesis that differ considerably in strength.^16^

First, Sullivan emphasizes the predictive accuracy of DNNs. The predictive accuracy of some DNNs is beyond dispute. Furthermore, it is very plausible that predictive accuracy is a necessary condition for understanding. If we want to provide explanations on the basis of DNNs, then the predictions need to be (approximately) correct. Most importantly, the DNN has to reproduce the *explanandum* phenomenon. Despite this, predictive success is not sufficient for explanatory understanding unless a predictively accurate tool provides at least some explanatory information.

The second achievement that Sullivan discusses regarding understanding with DNNs is how-possibly explanation. It is reasonable to require how-possibly explanations as a necessary condition for understanding. Still, this does not underwrite Sullivan’s claim that DNNs provide us with understanding about the target system, because it is dubious whether how-possibly explanations suffice for a significant degree of explanatory understanding. A how-possibly explanation provides a possible mechanism that reproduces the explanandum phenomenon; it does not need to provide the real mechanism that produced the phenomenon. A how-possibly explanation need not be an explanation.^17^

It is, admittedly, debatable whether explanatory understanding requires how-actually rather than how-possibly explanations. For Khalifa (2017), explanatory understanding has to get close to a correct explanation; de Regt (2017) allows that how-possibly explanations contribute to understanding, but only because they make the theory that is used more intelligible. All we need at this point, however, is the assumption that how-possible explanations provide a weaker form of understanding than how-actually explanations.

A different question is the extent to which DNNs do in fact provide how-possibly explanations. At least Sullivan’s own examples are not convincing. For instance, she writes that the deep patient model can answer the question of “how it is possible to predict disease development for a range of diseases” (Sullivan, 2022, p. 123). But this is not a request for a how-possibly explanation of phenomena in the target system, it is a question about the possibility of predictive modeling itself. Therefore, an answer to the question does not provide a how-possibly explanation about phenomena in the target system.

The third achievement stressed by Sullivan is that DNNs can produce understanding by facilitating further investigations by researchers. We interpret this as the claim that DNNs play a heuristic role. We agree that DNNs can do this and that this is a necessary condition for understanding. However, playing a heuristic role is an even weaker criterion for understanding than the previous two; in particular, DNNs can play a heuristic role without being empirically adequate. If the notion of understanding using DNNs boils down to playing a heuristic role, then the Negative Thesis is weak as well: DNNs merely need to contribute some useful information to the process of research, which is certainly the case today.

This is not to downplay the role of heuristics in science. They are a very important aspect of science, and do provide a kind of understanding. In fact, we believe that the heuristic role of ML in science in general deserves more attention (see Zednik and Boelsen, 2020 for examples). It is just important to be clear about what kind of understanding DNNs produce. Sullivan emphasizes the heuristic role of DNNs in the discussion of the three case studies, while the discussion of understanding with DNNs in the introduction of her paper is not qualified in this manner.

All in all, we have argued that all three achievements of DNNs used by Sullivan form necessary conditions for explanatory understanding or that they advance this kind of understanding to some measure, but that they do not lead to a high degree of explanatory understanding, because they are too far from actual explanations. The fact that DNNs attain these achievements to a degree only shows that we obtain understanding with DNNs in a weak sense. In this weak sense, the Negative Thesis not very interesting; for instance, it is plausible that DNNs can be fruitful heuristics for finding new candidate explanations. A stronger and more interesting reading of the Negative Thesis presumes that understanding of phenomena requires predictive accuracy *and* something close to how-actually explanations. This stronger reading is important because explanation is often taken to be among the goals constitutive of science (a point made e.g. by Aristotle, in the first book of his *Metaphysics*).

Our argument in this section is not meant to deny that DNNs can be helpful in producing some kind of understanding. In fact, we propose that DNNs can help to produce some degree of *objectual* understanding (see, e.g., Baumberger et al. 2017, Sect. 3 for a review). Objectual understanding is the understanding of a domain of things; it is often taken to imply some knowledge of this domain and the grasp of connections between items in the domain. These connections may be explanatory, but need not be; they may be merely logical or probabilistic (Kvanvig, 2003, pp. 191–192). As a consequence, there can be a degree of objectual understanding without an actual explanation. For instance, Gijsbers (2013) argues that a classification (e.g., using biological species) can enhance our understanding without explanation. ML models can lead to some objectual understanding, e.g. by establishing correlations, or by simply adding to knowledge of a domain of things.^18^ Consequently, Sullivan’s Negative Thesis is much more plausible when we take her to be talking about objectual understanding.^19^

### Lack of Understanding of DNNs Impedes Understanding With DNNs

We now turn to the crux of our argument against the Negative Thesis. We will show, first, that there are high-level properties of DNNs that we do not (currently) understand, and second, that this lack of understanding impedes our ability to understand phenomena using DNNs as a vehicle. Specifically, our lack of understanding of these properties means that we do not know why some DNNs are predictively successful, and that this, in turn, implies that we cannot use these DNN to obtain how-actually explanations about the target system, which is to say that our understanding of the target system with these DNNs is limited. Thus, a strong reading of the Negative Thesis is wrong.

First, we turn to properties of DNNs that we do not understand. In the computer science literature, several such properties are discussed—here is a first example. We have seen that some DNNs are very successful at solving a variety of problems. In particular, some DNNs generalize well. However, the reasons why these DNNs generalize well is only insufficiently understood (Zhang et al., 2017; Shwartz-Ziv & Tishby, 2017; Alain & Bengio, 2016; Zhang et al., 2021; Berner et al., 2021). Zhang et al. (2017) ask how we should characterize the difference between DNNs that generalize well and DNNs that do not, and they find that we do not have a satisfactory answer to this question. To support this claim, they consider several benchmark data sets on which DNNs perform well. They shuffle the labels attached to the images in the training sets in a random way, and train DNNs on these shuffled data sets. Surprisingly, they find that the DNNs are still able to perform well on the training sets. (They do not perform well on test sets, because the random shuffling prevents the models to learn anything generalizable from the data.) This means that DNNs just memorized the shuffled data. This is puzzling, because we know that the DNNs did not just memorize the original data; otherwise, they would not have been able to perform well after training on the original data sets. How is it that DNNs are able to extract a signal from the original data without overfitting, while also memorizing the randomized data, which amounts to overfitting? Zhang et al. (2017) argue that existing notions from statistical learning theory, such as VC dimension, Rademacher complexity, and uniform stability, which are supposed to capture when a model does neither underfit nor overfit the data, are unable to account for this difference. Since Zhang et al. (2017) proposed their argument, a lot of research has gone into answering this question, and progress has been made. Still, in an updated version of Zhang et al. (2017), the authors write: “Despite significant progress on theoretical understanding of deep learning in the past few years, a full mathematical characterization of the whole story remains challenging” (Zhang et al., 2021, Sect. 6.1.). Berner et al. (2021, p. 22) write that generalization is now better understood, but, for the most part, only for simplified models, e.g,. in the linear case. There is no clear, agreed-upon answer as of yet, so this is a property of DNNs that we do not sufficiently understand.^20^

Turning to the second part of our argument, this lack of understanding of DNNs impedes our ability to understand with DNNs as a vehicle. The problem is that, if we do not know why DNNs generalize well, that is, if we do not know the reasons for their predictive success, then at least some DNNs are predictively successful for the wrong reasons, which is to say that these models do not give us how-actually explanations of the phenomena under scrutiny.

To make the point that we need to understand DNNs in order understand with DNNs as a vehicle, let us first consider the case of a single DNN. Assume that we want to use that DNN to understand a target system, and that we have successfully trained the model, such that it generalizes well for a number of test cases. If we do not understand what kinds of features the model uses, it is possible that the model relies on unstable features, or artifacts in the data, and that the model may therefore perform poorly on new, untested data. In other words, it is possible that the predictions are not based upon those kinds of features that explain the predictions or regularities in the target system.

Let us give a concrete example of a DNN that is predictively successful for the wrong reasons, in that its success is based on spurious correlations.^21^ Spurious correlations are misleading about the causal relations underlying a prediction. If a model is based on spurious correlations, the model’s predictive accuracy may be bad for a relevant subset of data, namely the subset where the difference between mere correlation and causal relation makes an observable difference. Caruana et al. (2015) provide a notorious example of a model for hospital admission triage that is (at least to a large extent) predictively successful, but makes wrong and harmful predictions for a subset of cases. However, a model that is to a large extent predictively successful, but that may still be systematically wrong about a target system given the knowledge of the researchers, is not a good tool for yielding understanding about the target system.

Turning to the general case, why might a better understanding of generalization properties of DNNs help here? One way to understand generalization properties better is to prove an upper bound on the so-called generalization error (a measure of how well a DNN performs in the worst case), given assumptions about the data distribution, features of the model and of the learning algorithm. Theorems of this sort will allow us to identify DNNs that perform well even in the worst case, as opposed to other DNNs about which we do not know how well they perform in the worst case, even though they may perform well in empirical tests. This latter category is likely to include models that exploit spurious correlations, such as the case of hospital admission triage mentioned above. However, if a DNN provably generalizes well even in the worst case, it is likely that this model will not rely on spurious correlations, at least for the most part. So we can be more confident that the model isn’t successful for the wrong kinds of reasons.

Note that it may be possible to understand why a specific DNN performs well, without a general understanding of the generalization properties of DNNs. However, the fact that so many DNNs perform well suggests that there is a general reason or explanation for this phenomenon, which is likely to be provided by a general story of what kind of features DNNs use to predict successfully. Note also that even if we understand why one single DNN performs well, and this improves our ability to understand with this DNN as a vehicle, this is sufficient for our argument against the Negative Thesis.

At this point, it could be objected that we are running two things together. If a DNN picks up on spurious features, this seems like a matter of link uncertainty, that is, of how the model and the world are related, and not of our understanding the model itself. We reply: As pointed out above, researchers do not fully understand which features the DNN picks up on, nor how these features are combined to produce the final classification. However, understanding how this works means understanding how the model as such behaves in general, that is, how features are combined for any input, and not how the model relates to a particular dataset, and thus to its target. So what is missing here, we suggest, is understanding of the model itself.

There is a more general lesson to be learned at this point. What our discussion shows is that link uncertainty and understanding of DNNs cannot be separated in a clear-cut manner. They are not independent quantities, as it were; rather, if link uncertainty is removed, we will typically understand our model better. Conversely, given some background knowledge about the target, a better understanding of the DNN can reduce link uncertainty. The idea that understanding of the model and link uncertainty are independent is misguided. If this is so, the truth of the Positive Thesis undermines the truth of the Negative Thesis.

The problem of understanding the generalization properties of DNNs is one important open problem, but it is not the only one. Vidal et al. (2017) and Berner et al. (2021) note several further open theoretical problems. One important example concerns the optimization properties of DNNs: It is not known whether stochastic gradient descent (SGD) finds the best approximation $\hat{f}$ of a function *f* of interest. Vidal et al. (2017) report that there is much empirical and some theoretical evidence that local minima are mostly harmless, but the question is not yet settled. Again, our lack of understanding of this high-level property of DNNs limits our grasp of the predictive accuracy of our use of DNNs.

Our argument might easily be misunderstood as saying the following: the higher the accuracy of a DNN, the better our understanding obtained by using the DNN. Since we do not know the scope of the predictive accuracy of a DNN, we cannot render the DNN more accurate. Thus, our understanding of the phenomena using the DNN remains limited. On this reading, the argument seems pointless with regard to the Negative Thesis: It makes the rather trivial point that new and more accurate models would improve our understanding of the target. The Negative Thesis does not deny this. Rather, its point is that in the current situation, our poor current understanding of DNNs does not much limit their use in understanding real-world target systems.

Properly understood, however, our argument is in fact directed against the Negative Thesis: If a DNN is to be used to understand a phenomenon, it has at least to reproduce the phenomenon to some degree of accuracy. In this sense, a certain level of predictive accuracy is necessary for understanding. But merely reproducing the phenomenon is not sufficient for explanation. Rather, we need to understand how and why the model reproduces the phenomenon. For instance, we need to understand the scope of the predictive success of the model. We currently do not have this understanding of DNNs, so our understanding of phenomena using DNNs is impaired.

It may be objected that there are always properties of models that we do not understand, but that the impact on understanding with these models is very minor. Relatedly, it may be suggested that understanding is context- and audience-dependent, such that certain questions that we cannot answer about target systems using DNNs do not matter much, at least for certain stakeholders.^22^

We reply that, first, there are further, crucial aspects of DNNs that we do not understand (see our examples above and Vidal et al., 2017; Fischer, 2020). Second, independent of purposes, explanatory understanding (which is the focus of Sullivan’s paper) requires answers to explanatory questions (it may be different with objectual understanding). We have exhibited an important property of DNNs that we do not understand and that impedes explanatory understanding with DNNs. This suffices to reject the Negative Thesis. More generally, the problem about understanding that we have discussed above should worry those stakeholders who want to avoid predictions based on spurious correlations.

## Conclusion

Sullivan has argued that our understanding with DNNs is not limited by our understanding of the DNNs themselves (we have called this the Negative Thesis), but rather by link uncertainty (Positive Thesis). In this paper, we have argued that the plausibility of the Negative Thesis crucially depends on the assumed notion of understanding. The Negative Thesis comes out wrong if we use a strong reading of understanding with DNNs, notably if we focus on explanatory understanding and require something close to knowledge of a correct explanation for this variety of understanding. The Negative Thesis is more plausible if we focus on a weaker notion of understanding, e.g. a sort of objectual understanding. Thus, we have to be precise about the notions of understanding *of* DNNs and understanding *with* DNNs. More philosophical work is needed to spell out both notions and to learn about their mutual relationships.

## Footnotes

1 See, e.g., Zhang et al. (2017), Shwartz-Ziv and Tishby (2017), Alain and Bengio (2016), Zhang et al. (2021), Berner et al. (2021) and Sect. 5.2.

2 Zerilli et al. (2019) are also critical of the demand for transparent algorithms; however, their focus is not on the scientific use of ML, but rather on decision making of ML models more broadly.

3 Sullivan mostly focuses on a simple variety of DNNs: fully connected feedforward neural networks in a supervised learning setting. We will also restrict attention to this kind of ML model.

4 See, e.g., Sullivan (2022, p. 110) : “[I]t is the level of ‘link uncertainty’ present – that is, the extent to which the model fails to be empirically supported and adequately linked to the target phenomena – that prohibits understanding”.

5 Some of Sullivan’s formulations suggest a stronger thesis, according to which understanding ML models is not at all necessary for understanding with ML models, for example: “model simplicity and transparency are not needed for understanding phenomena” (Sullivan, 2022, p. 110). Our argument against Sullivan’s claim in the weaker sense suggests that it is wrong in its stronger reading too.

6 The discussion of understanding with climate models in Jebeile et al. (2021) suggests “empirical accuracy”, which corresponds to predictive accuracy, as one evaluative criterion for understanding with DNNs.

7 The question of whether how-possibly explanations provide understanding has been discussed in the literature, see, e.g., Rice (2016), Verreault-Julien (2019).

8 The exploratory role of ML models, in combination with xAI techniques such as visualizations, is highlighted in Zednik (2021).

9 The following discussion of MNIST is based on Nielsen (2015); a more thorough account of supervised learning can be found there or in Hastie et al. (2009) and in Goodfellow et al. (2016).

10 For the sake of the argument, we assume that we (or the experts) agree about *y*.

11 For some DNNs, the classification is correct in over 99% of the data in $X'$; see Nielsen (2015).

12 See Wilkenfeld (2019) for an articulation of the idea that having a short, compressed representation of an object, and being able to use this representation, is key to understanding.

13 Here we gloss over the fact that the set of pictures of a handwritten digit *S* is a subset of all black-and-white pictures of this format, *P*; the subset *S* is many orders of magnitude smaller than *P*. However, even if *S* is tiny in comparison to *P*, it is still much too big to classify its elements by hand. Additionally, it is probably very hard to obtain or describe *S*. We also gloss over the fact that the DNN does not really implement *f*, because the DNN does not fully reproduce *f*, but merely approximates *f*.

14 See Lipton (2018), Doshi-Velez and Kortz (2017), Ribeiro et al. (2016).

15 Zednik (2021) proposes the use of heat maps to answer questions about *why* an ML model does what it does, which yields understanding with the model. However, as he acknowledges, sometimes, “the utility of input heatmapping is likely to be limited” (ibid., p. 277) and “there is also considerable room for improvement” (ibid., p. 286). The adequacy of so-called xAI techniques, which encompass saliency maps, has been repeatedly questioned in the computer science literature; see, e.g., Rudin (2019).

16 Note that our three readings of the Negative Thesis arise because we take the notion of understanding *with* ML models to be unclear. It may also be asked what exactly understanding *of* ML models means. For our discussion this does not matter much, because whatever understanding *of* ML models is, the degree of this understanding is fairly low. We take it that Sullivan’s main point is to argue that despite this comparably poor understanding of DNNs, they can help us to understand real-world phenomena.

17 For instance, there are agent-based computer simulations that reproduce facts about the history of the Anasazi community (Grüne-Yanoff, 2009). However, if the assumptions underlying the simulation do not capture the interactions among agents that led to the historical development, the simulations do not explain the history, which means that the simulation does not much advance our explanatory understanding.

18 Since there are many questions the answers to which contribute to objectual understanding, the degree to which objectual understanding is advanced depends on how important the various questions are. This, in turn, depends on the purposes of the agent, so objectual understanding is purpose-dependent. The purpose-dependence of understanding is also stressed by Zednik (2021), who does not, however, discuss objectual understanding.

19 There is, maybe, another ambiguity in the discussion: It is not clear what it means to say that a real-world target phenomenon is understood (qua explanatory or qua objectual understanding) *using*/*with*/*with the help of* some tool T. Theories and mathematical models help us to understand a phenomenon because they contain the relevant connections. In particular, explanations are basically inferences from theories (and perhaps from auxiliary information and modeling assumptions). Other tools act more indirectly to enhance our understanding, e.g., because the results obtained using the tools suggest explanatory hypotheses, which can then be examined in detail. It is plausible to say that ML models and DNNs can help us to understand phenomena in this more indirect sense. But ML models do not help us to understand phenomena in the way theories and some other models do. Regarding theories, this point follows from the account of scientific understanding by de Regt (2017). For him, it is a necessary requirement that the theory used be intelligible to the researcher, and this is not the case with ML models, at least with many DNNs.

20 Proposals to explain the successes of DNNs have been discussed in the recent philosophical literature; see Buckner (2018, 2019) for an analysis of why so-called convolutional neural networks generalize well, and Räz (2022) for a statistical explanation of the successes of DNNs; see also Zhang et al. (2021), Berner et al. (2021) and the many citations of Zhang et al. (2017) in the computer science literature.

21 The fact that uncovering correlations is not sufficient to uncover causal structures is well known, see Spirtes et al. (2000) and Pearl (2009).

22 See, e.g., Zednik (2021) for a discussion of different stakeholders’ explanatory requirements in explainable AI.
